# Trailblazing through the Opioid Epidemic. Will Science Prevail?

**Published:** 2023-06-20

**Authors:** Panop Limratana, Koichi Yuki

**Affiliations:** 1 Department of Anesthesiology, Critical Care and Pain Medicine, Boston Children’s Hospital, Boston, USA; 2 Department of Anaesthesia, Harvard Medical School, Boston, USA; 3 Department of Immunology, Harvard Medical School, Boston, USA; 4 Department of Anesthesiology, Faculty of Medicine Siriraj Hospital, Mahidol University, Bangkok, Thailand; 5 Broad Institute of Harvard and MIT, USA

The opioid epidemic among children and adolescents in North America is a growing concern. Between 1999 and 2018, 8,986 children and adolescents lost their lives due to opioid overdose, resulting in a rising mortality rate from 0.21 to 0.81 per 100,000 children/year [1]. The recent COVID-19 pandemic has further exacerbated the situation by impeding the development of new treatments and patient access to healthcare [2,3]. Given the magnitude of the crisis, there is an immediate need for the development of safer opioids and alternative interventions to minimize opioid-related deaths. In this article, we will explore three promising discoveries targeting different mechanisms of opioid adverse effects, particularly among the pediatric population.

A notable and direct solution to reduce opioid-related mortality is to develop opioids that are free of respiratory side effects. Recently, oliceridine, a novel mu-opioid receptor (MOR) agonist, has been approved by the Food and Drug Administration for the treatment of moderate to severe pain [4]. In contrast to the conventional opioids with both G-protein and β-arrestin agonism, oliceridine exerts its effect on the G-protein coupled pathways (biased agonism). This is hypothesized to minimize respiratory depression effects. Randomized trials comparing oliceridine and conventional opioids demonstrated its analgesic efficacy and superior safety profiles, particularly in protecting against respiratory depression at clinical doses [5,6]. Although oliceridine studies were largely based on the notion of biased agonism, the true mechanisms of oliceridine are yet to be elucidated. Studies in β-arrestin knocked-out mice showed that opioid side effects are likely due to G-protein-mediated pathways, contradicting the theory of biased agonism. From the pharmacodynamic standpoint, oliceridine might essentially be a partial agonist with low intrinsic efficacy, thus reducing side effects [7]. In theory, oliceridine has potential benefits for the pediatric population, which is particularly vulnerable to opioid side effects. Additional clinical trials focusing on its safety and efficacy in this population are still warranted. Another potential pharmacologic approach is to prevent MORs from inducing respiratory depression effects.

Animal studies demonstrated that specific groups of MORs in the preBötzinger complex (preBötC) and the parabrachial nucleus (PBN) are largely responsible for opioid-induced respiratory depression (ORID), with PBN being the major contributor. MOR agonists developed specifically to be devoid of these specific MORs interactions will likely be free of ORID. However, due to the lack of pharmacologic specificity of these MORs, although attractive, it is currently impossible [8].

Researchers have been looking for new reversal agents secondary to the increasing number of fatal opioid overdoses and reports of intranasal naloxone’s limited half-life and efficacy in reversing severe ORID [9]. Drugs with respiratory stimulant effects have been extensively researched as naloxone alternatives, although with limited success in clinical implications [10]. Another novel approach targeting fentanyl-related ORID is by accelerating its elimination. Although fentanyl’s half-life is relatively short, the elimination is dose-dependent and its context-sensitive half-time can be significantly long. To overcome this pharmacologic characteristic, an alternative pathway has been investigated. A recent study [11] in rats has shown that Calabadion 1 (CLB1), a novel container molecule, can selectively encapsulate free-form fentanyl in circulation, similarly to what sugammadex does to rocuronium. In this randomized placebo-controlled trial [11], CLB1 treatment resulted in the dose-dependent reversal of ORID in anesthetized rats. Following encapsulation, the CLB1-fentanyl complexes can be readily excreted by the kidney, thus avoiding the re-narcotization seen with naloxone. Another unique property of CLB1 is the affinity to fentanyl (100-fold) compared to morphine. This highly specific binding could benefit perioperative use when fentanyl is combined with other long-acting opioids. In this scenario, CLB1 can reverse ORID from fentanyl without reversing the analgesic effects of other opioids such as morphine, mitigating the risk of inducing unwanted pain. CLB1 might be of limited value in the event of non-hospitalized, life-threatening ORID where immediate reversal of unknown opioids is necessary. Further trials are still needed before CLB1 will be available for clinical use.

An opioid vaccine is another promising intervention that could reduce opioid-related mortality and could be one of the most effective interventions. Researchers have recently developed anti-fentanyl vaccines and successfully tested them on animals. This immunotherapy approach is an alternative intervention to target opioid use disorder which currently depends mainly on pharmacotherapeutics that require patient compliance and long-term monitoring [12]. The proposed mechanism is that the anti-fentanyl IgG could bind to fentanyl in the circulation, creating the fentanyl-antibody complexes that cannot cross the blood-brain barrier. The results are diminished analgesic and behavioral effects, and prevention of ORID [13]. In a recent study [14], researchers gave rats three fentanyl-antigen vaccine injections (3-week intervals) that resulted in the detection of anti-fentanyl IgG antibodies in blood samples four weeks later. In the tenth week after immunization, vaccinated and non-vaccinated rats were injected with high-dose fentanyl. Non-vaccinated rats developed profound respiratory depression, while vaccinated rats did not. Additionally, brain fentanyl concentrations were significantly higher in the non-vaccinated rats confirming the hypothesis of fentanyl-IgG complexes and blood-brain barrier permeability. In a related study [15], researchers utilized carfentanil, a highly potent synthetic opioid, structurally similar to fentanyl, and developed vaccines that induce antibody production in mice. The vaccinated mice exhibited attenuated behavioral effects as well as lower brain concentrations of fentanyl compared to the control. These protective effects were still evident at eighty days after immunization, indicating the long half-life of the antibodies. Undoubtedly, opioid vaccines can significantly supplement the treatment of opioid use disorders in the future. This is particularly advantageous in adolescents because the difficulty in accessing treatments for opioid use disorders [16].

With these promising approaches in mind, we still have to consider the disparity between the availability of new drugs for adults and children that has existed for a long time [17]. Drugs are typically tested in adults first prior to the pediatric population. Historically, approximately ten years are the average amount of time for a drug approved in the adult population to receive approval for use in the pediatric [18]. However, legislation such as the Pediatric Research Equity Act (PREA) and the Best Pharmaceuticals for Children Act (BPCA) hopefully helps to facilitate studies of future drugs to intervene opioid epidemic in pediatric populations. In case of COVID-19 vaccine development, it was an extremely fast approval time for pediatric use.

Opioid crisis is a complex and multifaceted problem requiring comprehensive solutions. Clinically, optimizing opioid prescriptions can be readily performed. Screening for the risks of opioid misuse should be routine before prescribing to adolescents. Patients and families should be educated on the benefits and risks of opioids. Means of safekeeping and discarding unused pills should be clearly communicated [19]. Comprehensive educational programs involving adolescents, teachers, and healthcare providers are available and should be promoted. Concurrently, bench-to-clinical translation research, drug discoveries, and novel treatment modalities can all be keys to overcoming the opioid crisis. As seen in the COVID-19 vaccine development, scientific progress has advanced at an unprecedented pace. With continued ingenuous efforts, we can hope to conquer this battle.

## References

[1] Gaither JR, Shabanova V, Leventhal JM. US national trends in pediatric deaths from prescription and illicit opioids, 1999–2016. JAMA Netw Open. 2018;1(8):e186558. 30646334 10.1001/jamanetworkopen.2018.6558 PMC6324338

[2] Kosten TR, Petrakis IL. The hidden epidemic of opioid overdoses during the coronavirus disease 2019 pandemic. JAMA Psychiatry. 2021;78(6):585–6. 33377967 10.1001/jamapsychiatry.2020.4148

[3] Imtiaz S, Nafeh F, Russell C, Ali F, Elton-Marshall T, Rehm J. The impact of the novel coronavirus disease (COVID-19) pandemic on drug overdose-related deaths in the United States and Canada: a systematic review of observational studies and analysis of public health surveillance data. Subst Abuse Treat Prev Policy. 2021;16(1):87. 34844624 10.1186/s13011-021-00423-5 PMC8628272

[4] Lambert D, Calo G. Approval of oliceridine (TRV130) for intravenous use in moderate to severe pain in adults. Br J Anaesth. 2020;125(6):e473–e4. 33070948 10.1016/j.bja.2020.09.021 PMC7560257

[5] Simons P, van der Schrier R, van Lemmen M, Jansen S, Kuijpers KWK, van Velzen M, Respiratory effects of biased ligand oliceridine in older volunteers: A pharmacokinetic-pharmacodynamic comparison with morphine. Anesthesiology. 2023;138(3):249–63. 36538359 10.1097/ALN.0000000000004473

[6] Singla NK, Skobieranda F, Soergel DG, Salamea M, Burt DA, Demitrack MA, APOLLO-2: A randomized, placebo and active-controlled phase III study investigating Oliceridine (TRV130), a G protein-biased ligand at the μ-Opioid receptor, for management of moderate to severe acute pain following abdominoplasty. Pain Pract. 2019;19(7):715–31. 31162798 10.1111/papr.12801 PMC6851842

[7] Gillis A, Kliewer A, Kelly E, Henderson G, Christie MJ, Schulz S, Critical assessment of g protein-biased agonism at the μ-opioid receptor. Trends Pharmacol Sci 2020;41(12):947–59. 33097283 10.1016/j.tips.2020.09.009

[8] Ramirez J-M, Burgraff NJ, Wei AD, Baertsch NA, Varga AG, Baghdoyan HA, Neuronal mechanisms underlying opioid-induced respiratory depression: Our current understanding. J Neurophysiol. 2021;125(5):1899–919. 33826874 10.1152/jn.00017.2021 PMC8424565

[9] Dietze P, Jauncey M, Salmon A, Mohebbi M, Latimer J, van Beek I, Effect of intranasal vs intramuscular naloxone on opioid overdose: A randomized clinical trial. JAMA Netw Open. 2019;2(11):e1914977–e. 31722024 10.1001/jamanetworkopen.2019.14977 PMC6902775

[10] Dahan A, van der Schrier R, Smith T, Aarts L, van Velzen M, Niesters M. Averting opioid-induced respiratory depression without affecting analgesia. Anesthesiology. 2018;128(5):1027–37. 29553984 10.1097/ALN.0000000000002184

[11] Thevathasan T, Grabitz SD, Santer P, Rostin P, Akeju O, Boghosian JD, Calabadion 1 selectively reverses respiratory and central nervous system effects of fentanyl in a rat model. Br J Anaesth. 2020;125(1):e140–e7. 32241547 10.1016/j.bja.2020.02.019 PMC7418560

[12] Wakeman SE. Opioid use disorder diagnosis and management. NEJM Evidence. 2022;1(4):EVIDra2200038. 10.1056/EVIDra2200038 38319203

[13] Raleigh MD, Baruffaldi F, Peterson SJ, Le Naour M, Harmon TM, Vigliaturo JR, A fentanyl vaccine alters fentanyl distribution and protects against fentanyl-induced effects in mice and rats. J Pharmacol Exp Ther. 2019;368(2):282–91. 30409833 10.1124/jpet.118.253674 PMC6346379

[14] Haile CN, Baker MD, Sanchez SA, Lopez Arteaga CA, Duddupudi AL, Cuny GD, An immunconjugate vaccine alters distribution and reduces the antinociceptive, behavioral and physiological effects of fentanyl in male and female rats. Pharmaceutics. 2022;14(11). 10.3390/pharmaceutics14112290 PMC9694531 36365109

[15] Eubanks LM, Blake S, Natori Y, Ellis B, Bremer PT, Janda KD. A highly efficacious carfentanil vaccine that blunts opioid-induced antinociception and respiratory depression. ACS Chem Biol. 2021;16(2):277–82. 33533592 10.1021/acschembio.1c00026 PMC8260150

[16] Wu LT, Zhu H, Swartz MS. Treatment utilization among persons with opioid use disorder in the United States. Drug Alcohol Depend. 2016;169:117–27. 27810654 10.1016/j.drugalcdep.2016.10.015 PMC5223737

[17] Coppes MJ, Jackson C, Connor EM. I-ACT for Children: Helping close the gap in drug approval for adults and children. Pediatr Res. 2022:1–2. 10.1038/s41390-022-02349-5 PMC9589790 36271160

[18] Sachs AN, Avant D, Lee CS, Rodriguez W, Murphy MD. Pediatric information in drug product labeling. JAMA. 2012;307(18):1914–5. 22570457 10.1001/jama.2012.3435

[19] Kharasch ED, Clark JD, Adams JM. Opioids and public health: The prescription opioid ecosystem and need for improved management. Anesthesiology. 2022;136(1):10–30. 34874401 10.1097/ALN.0000000000004065 PMC10715730

